# Supplementary material for: Nuclear export of the pre-60S ribosomal subunit through single nuclear pores observed in real time
Source: Nat Commun. 2021 Oct 27;12:6211. doi: 10.1038/s41467-021-26323-7 (PMC8551241; doi:10.1038/s41467-021-26323-7)
Supplement: Supplementary file 1 — Supplementary Information [file 41467_2021_26323_MOESM1_ESM.pdf]

Supplemental Material

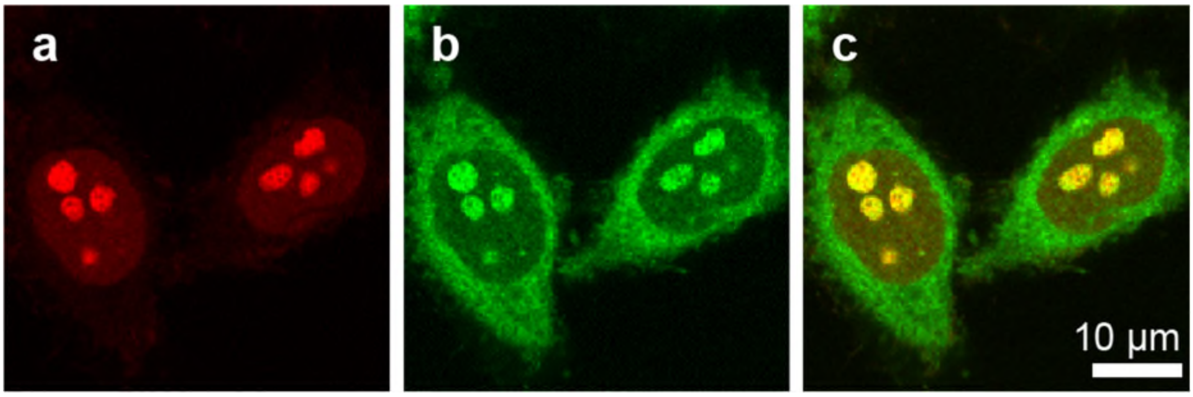

Supplemental Figure 1

eIF6-HaloTag-JF549 shows the same nucleolar distribution as Pno1-Snap-SiR647[1,2]. (a) HeLa cells expressing Snap-Pno1 stained with 647-SiR (red). (b) Transiently expressed eIF6-HaloTag stained with JF549 (green). (c) Overlay of both channels.

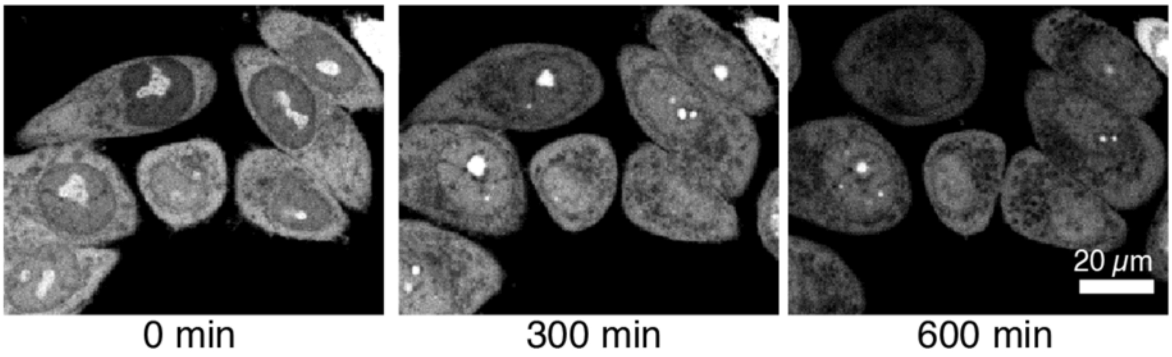

Supplemental Figure 2

Actinomycin D inhibits synthesis of new transcripts and leads to a loss of nucleolar staining implying that no further subunits were formed. Confocal sections of HeLa S3 cells stably expressing eIF6-HaloTag labelled by JF549 upon addition of 10 mM Actinomycin D. Images were taken subsequent to Actinomycin D addition: (a) 0 min, (b) 300min, (c) 600 min.

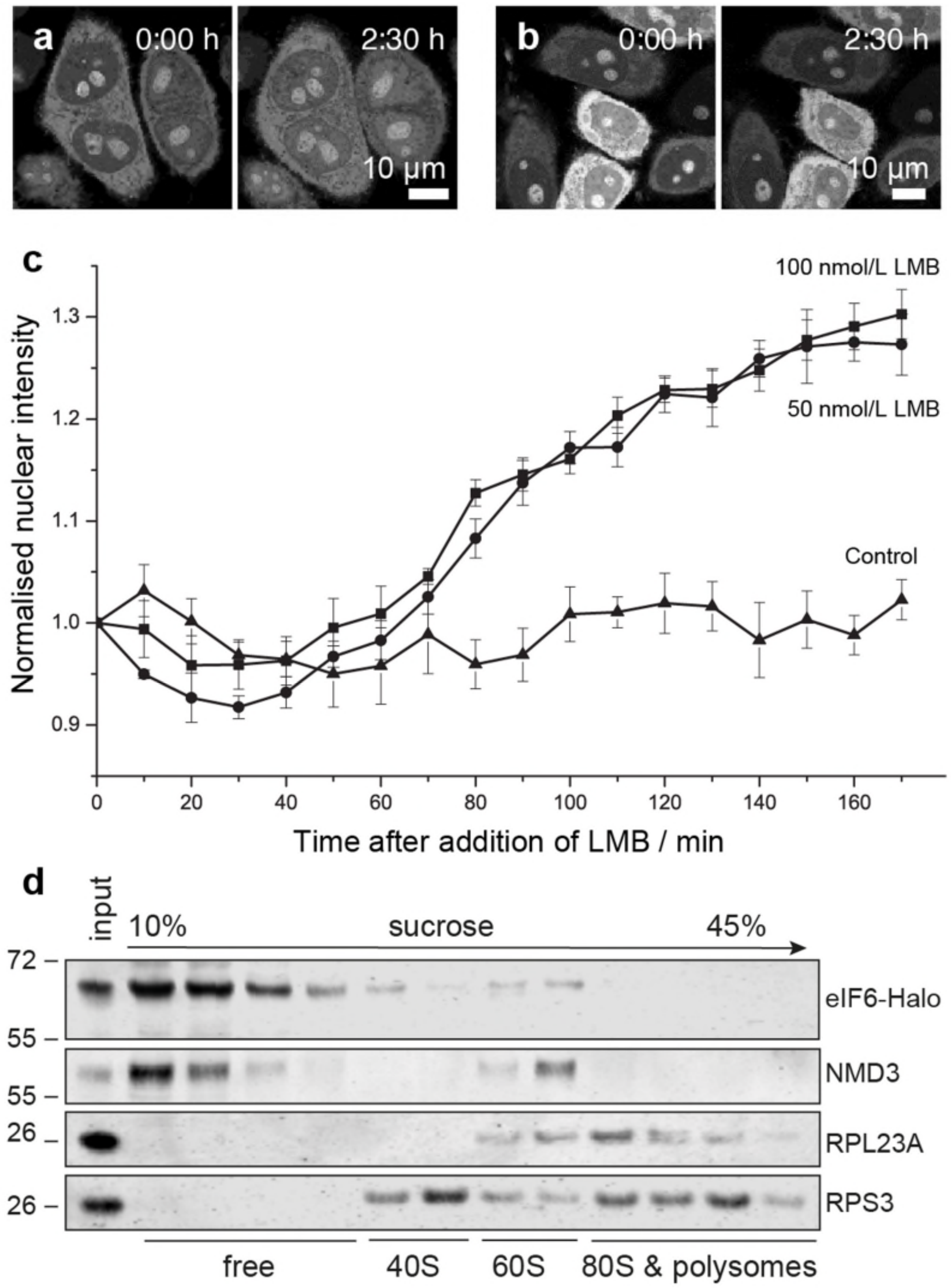

Supplemental Figure 3

LMB prevents Crm1 binding and thus nuclear export of the pre-60S subunit leading to an increase of intranuclear fluorescence. (a) eIF6-HaloTag-JF549 at 0:00 h and after 2:30 h incubation with 100 nmol/L LMB. (b) eIF6-HaloTag-JF549 at 0:00 h and after 2:30 h incubation with 10 µL ethanol as control. (c) Normalised fluorescence intensity in the nucleus as a function of time at a concentration of 50 nmol/L LMB (N=7, circles), a concentration of 100 nmol/L (N=9, squares) and a control measurement with ethanol (N=7, triangles). Error bars represent the standard error of the mean. (d) Extract of HeLa cells expressing EIF6-HaloTag cells was separated on a 10-45% sucrose gradient by centrifugation. Input and gradient fractions were analyzed by immunoblotting using the indicated antibodies. (NMD3 and RPS2 from [3], RPL23A from [4] and eIF6-Halo from [5])

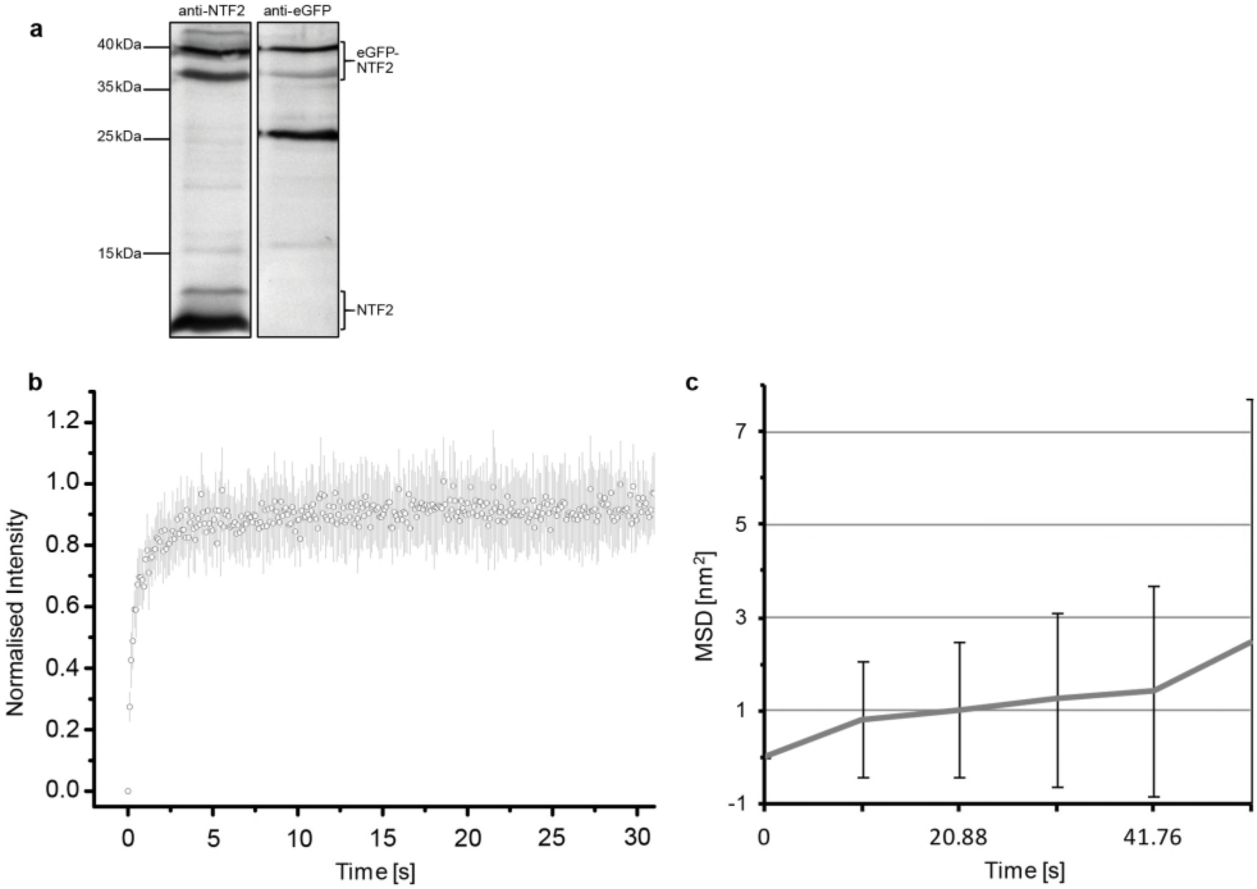

Supplemental Figure 4

(a) Western blot of the full lysate of HeLa-S3 cells stably expressing eGFP-NTF2. 5 µL of the full lysate were applied to each of the gel columns. Proteins were separated by electrophoresis in a 15% SDS-PAGE-Gel and then transferred to an Immune-Blot Nitrocellulose Membrane (Bio-Rad, Hercules, CA, USA) at 20 V and 0.3 A for 35 minutes. Membranes were blocked over night at 4°C with 3% milk powder in TBS-T (Tris-buffered saline, 0.1% Tween20). Next, membranes were incubated first with anti-NTF2 (1:200 in 3% milk powder in TBS-T, NTF2 Monoclonal Antibody, #sc-271693 (Lot: #B1914), Santa Cruz Biotechnology) or anti-GFP antibody (1:500 in 3% milk powder in TBS-T, eGFP Monoclonal Antibody F56-6A1.2.3, #MA1-952 (Lot: #TE260472), Thermo Fisher, Waltham, MA, USA) for 2h, washed 3x for 10 min with TBS-T and incubated with HRP antibody (1:500 in 3% milk powder in TBS-T, Goat anti-Mouse IgG (H+L) Secondary Antibody, HRP, #31430 (Lot: #UJ293428), Thermo Fisher) for 2h at RT. The blot was developed using 1-Step Ultra TMB Blotting Solution (Thermo Scientific) using manufacturer's instructions.

(b) FRAP curves were acquired from the eIF6-Halo/eGFP-NTF2 expressing cells kept under the same conditions like for the single molecule experiments. FRAP experiments were performed using the Zeiss LSM 880 microscope with a 25x/0.8 water immersion objective. A circular bleach spot (diameter 1 µm) was used for bleaching the nuclear membrane and 5 pre-bleach and 350 images after bleaching (90ms/frame) were recorded. An averaged, normalized curve from 10 single FRAP measurements is shown (circles) including the standard deviation of the data points (grey line). The estimated recovery time is 0.2 to 0.4 s and the mobile fraction is 90 to 95% (data are not fitted since diffusion fit models did not describe the combined diffusion/binding at the nuclear envelope well enough).

(c) Mean square displacement (MSD) of the NTF2 positions in living cells as a function of time: confocal Airyscan images of HeLa S3 cells stably expressing eGFP-NTF2 with alignment beads attached were acquired as described in the Methods section. The images were aligned using the alignment beads employing the StackReg plug-in for ImageJ. Pore positions were determined for each time point (see Methods) and the square displacement to the initial position was calculated (grey line) using OriginPro 8 (OriginLab Corporation, version v8.0988). The error bars correspond to the standard deviation of the data points. 253 NPCs in 9 cells were evaluated.

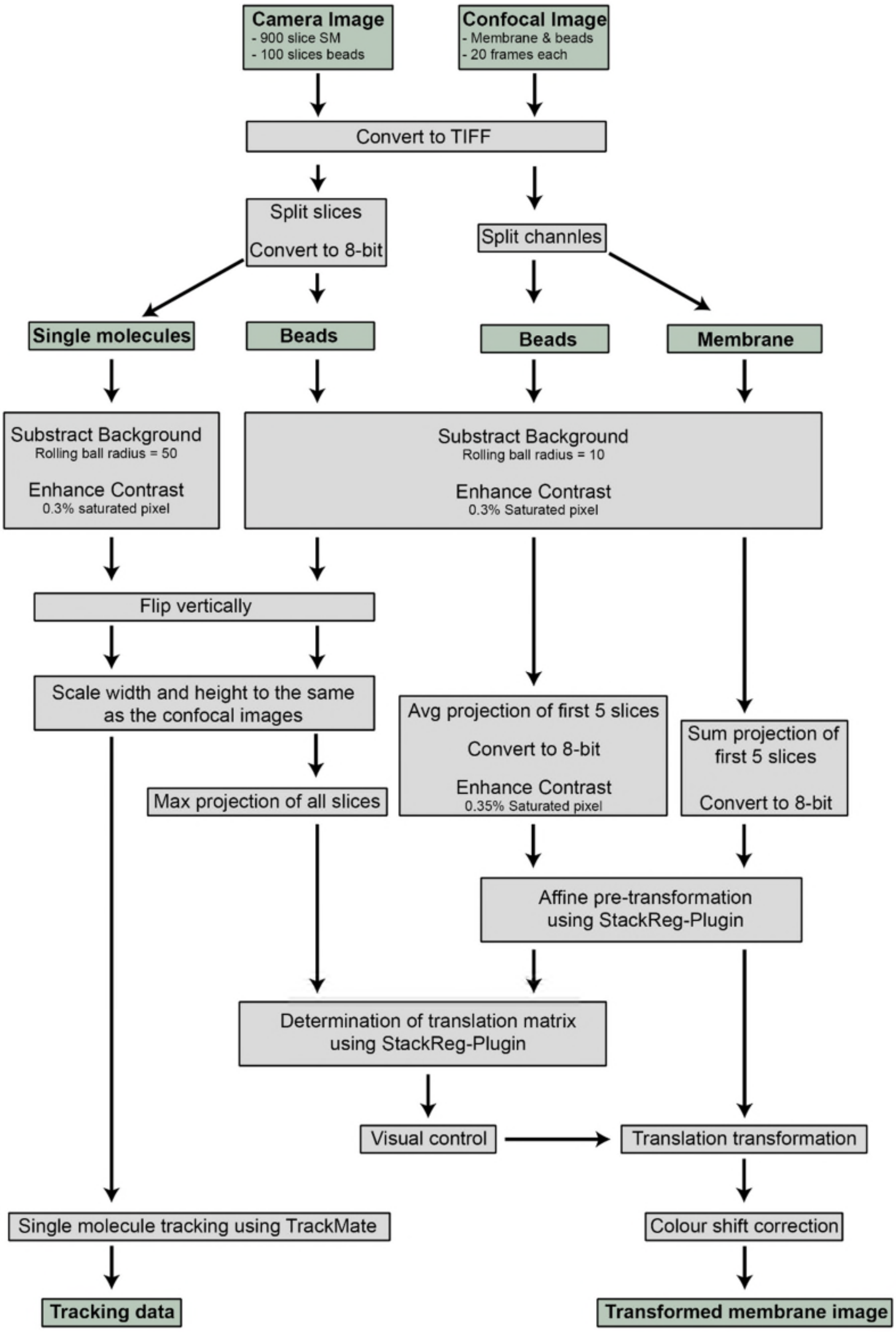

Supplemental Figure 5

Registration of images showing pre-60S particles and nuclear envelopes acquired with an EMCCD and a LSM 880 Airyscan, respectively. First, a movie of 1000 frames was acquired by the EMCCD camera. 900 frames were imaged with 561 nm excitation revealing the pre-60S subunits and 100 frames with 405 nm showing the reference beads. Next, 20 images were acquired using the LSM 880 Airyscan with alternating laser excitation (405 nm and 488 nm). Image processing of the EMCCD camera images: Image sequences were converted '8-bit TIFF', split into 900 frames of pre-60S subunits and 100 frames reference beads. Both image stacks were background-subtracted, contrast-enhanced and vertically flipped. A maximum intensity projection was calculated from the

reference bead stack. Single particles were tracked using TrackMate plugin for ImageJ. Image processing of the confocal images: Airyscan images were processed and converted into TIFF format. The two channels were separated, background-subtracted and contrast-enhanced. The first five frames of the reference beads were averaged and the contrast was again enhanced. The first five frames of the membrane image were summed. Both projections were converted to 8-bit. To align the EMCCD camera and Airyscan images, an affine transformation was applied using the StackReg plug-in for ImageJ to correct for rotation, shear and – roughly - the translation. To correct for stage drift during the measurement a further transformation matrix was determined from the bead images and applied to the Airyscan image of the NE. Finally, images were colour shift corrected.

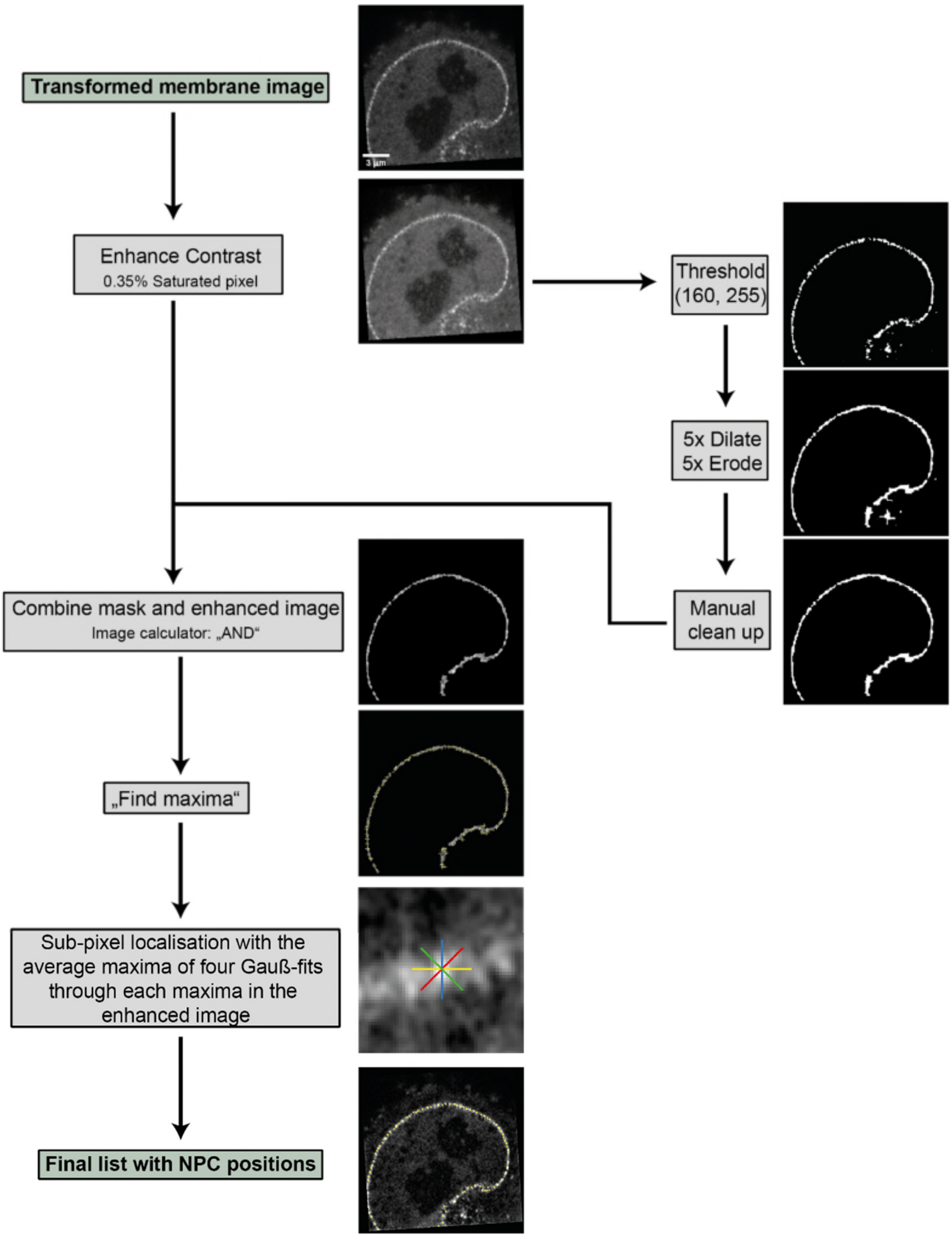

Supplemental Figure 6

Determination of single NPC positions. The coloured lines indicate the directions along which the fits were performed. For details, see Methods.

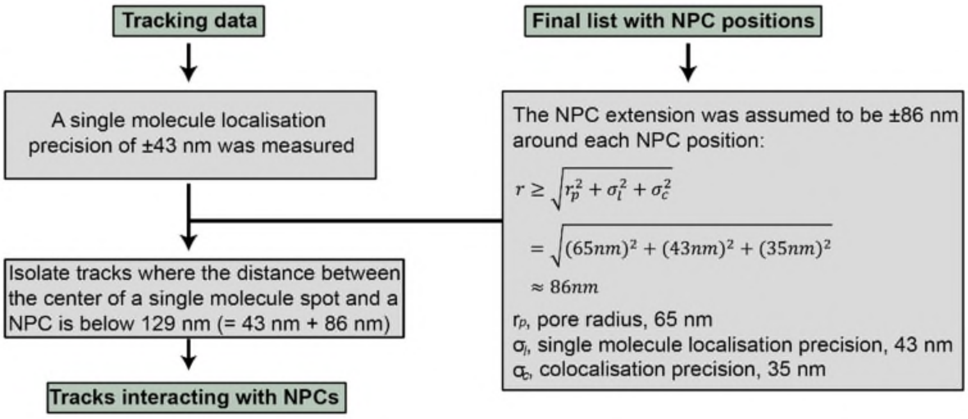

Supplemental Figure 7

Identification of single pre-60S particle tracks, which interacted with single NPCs. For details, see Methods.

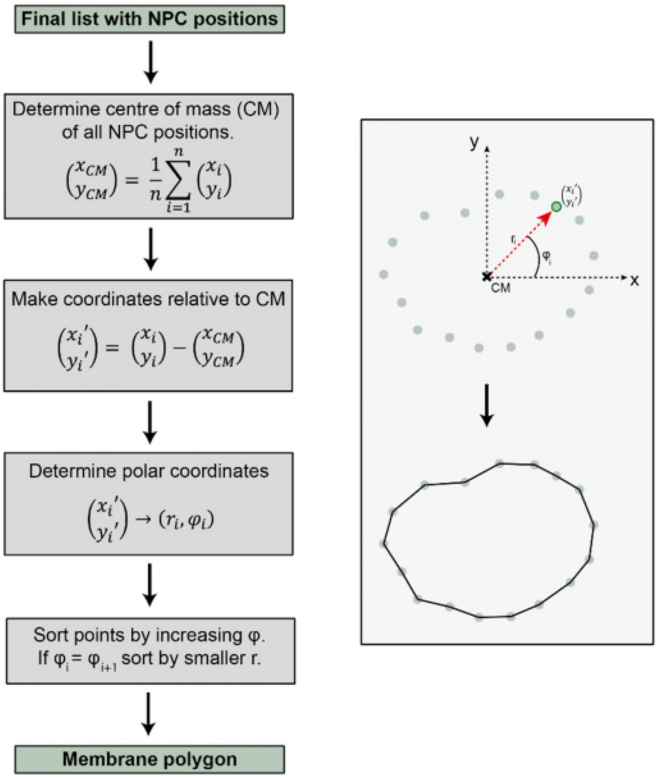

Supplemental Figure 8

Derivation of the polygon, which approximated the shape of the cell nucleus. For details, see Methods.

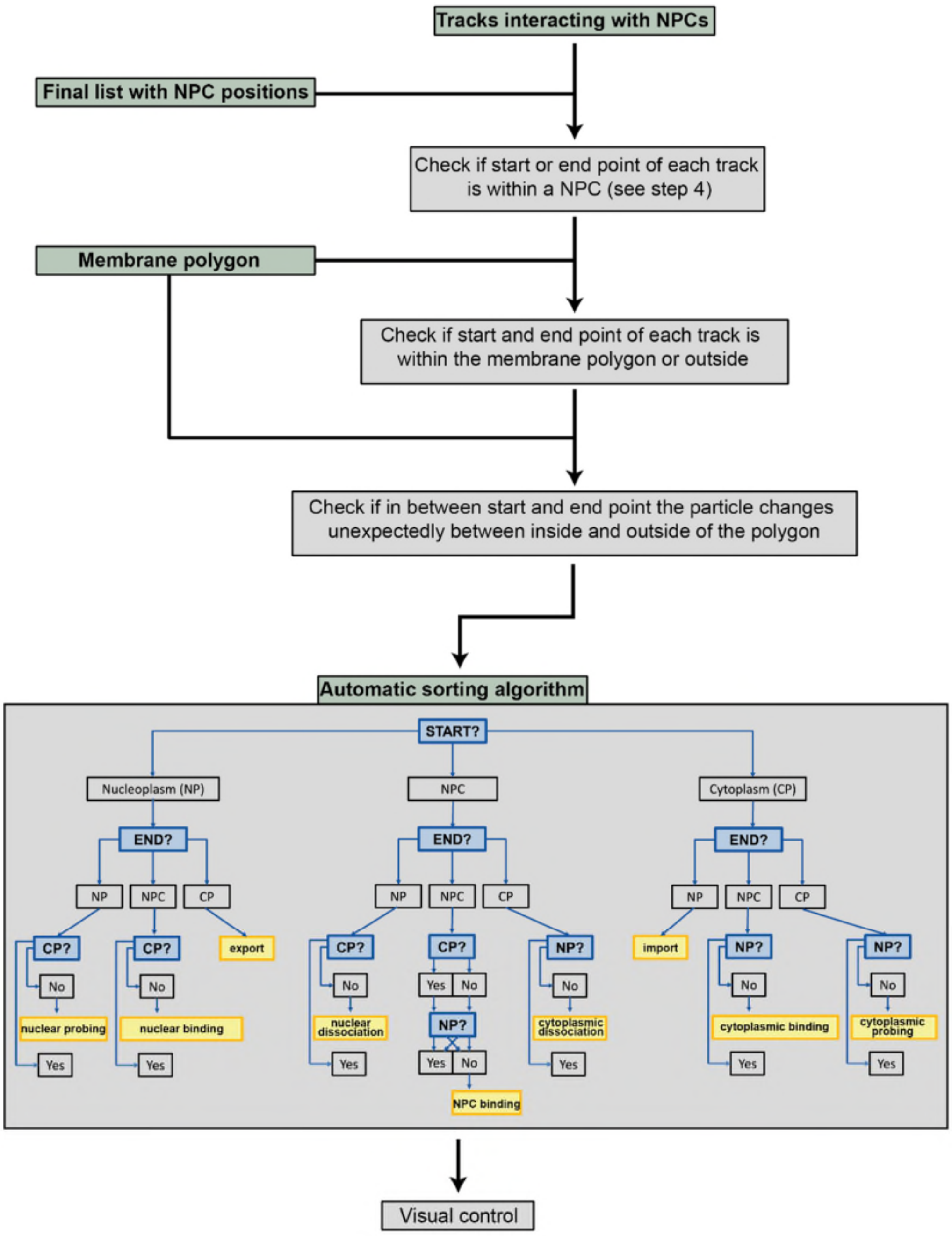

Supplemental Figure 9

Automated sorting of the observed trajectories interacting with single NPCs into the various contact modes as defined by Fig. 3g. For details, see Methods.

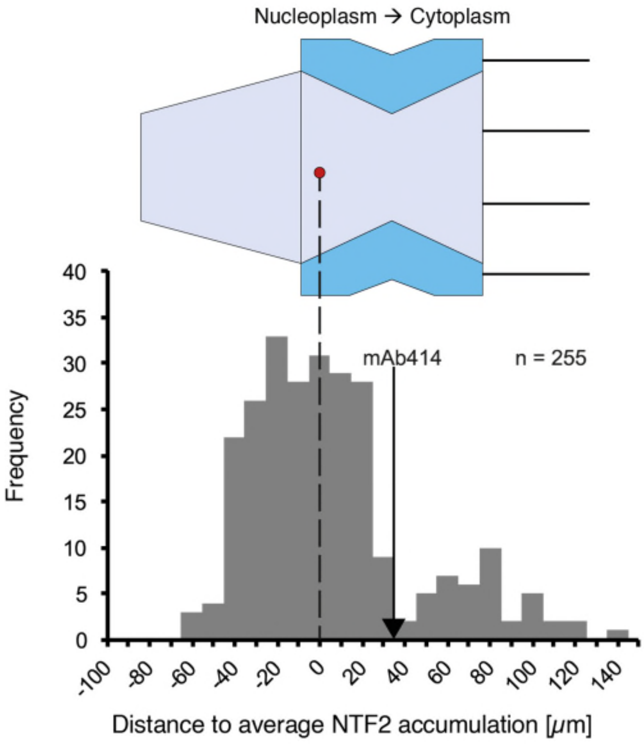

Supplemental Figure 10

Maximum NTF2 fluorescence intensity as indicator for the NPC center location, which was calibrated using the anti-nucleoporin p62 (Nup62) antibody mAB414 as reference. For n=255 NPCs in 33 cells the positions of the HaloTag-NTF2-JF549 and the binding sites of mAb414 (labelled by a polyclonal anti-mouse secondary antibody marked by AlexaFluor 488) maxima were determined by fitting four Gaussian functions in 45° angles (see supplemental Fig. 5). The maxima of the fits were averaged and the distance between the maxima of the Nup62 and NTF2 was calculated. The final distances were corrected by 5 nm to account for the mAb414 binding site maximum (see arrow) along the transport axis (shifted towards the cytoplasm with regard to the central plane of the NPC) as determined from localizations of mAb414 labeled by gold-anti-mouse antibodies in ultrathin sections of rat liver tissue by transmission electron microscopy (see Fig. 6g in Grote et al., 1995[6,7]) and shifted the whole histogram so that the average NTF2 maximum was at position 0. The average distance of NTF2 from the central plane of the pore is about -29 nm. Interestingly, we observed two maxima of the binding of NTF2, as has been observed previously[8–10].

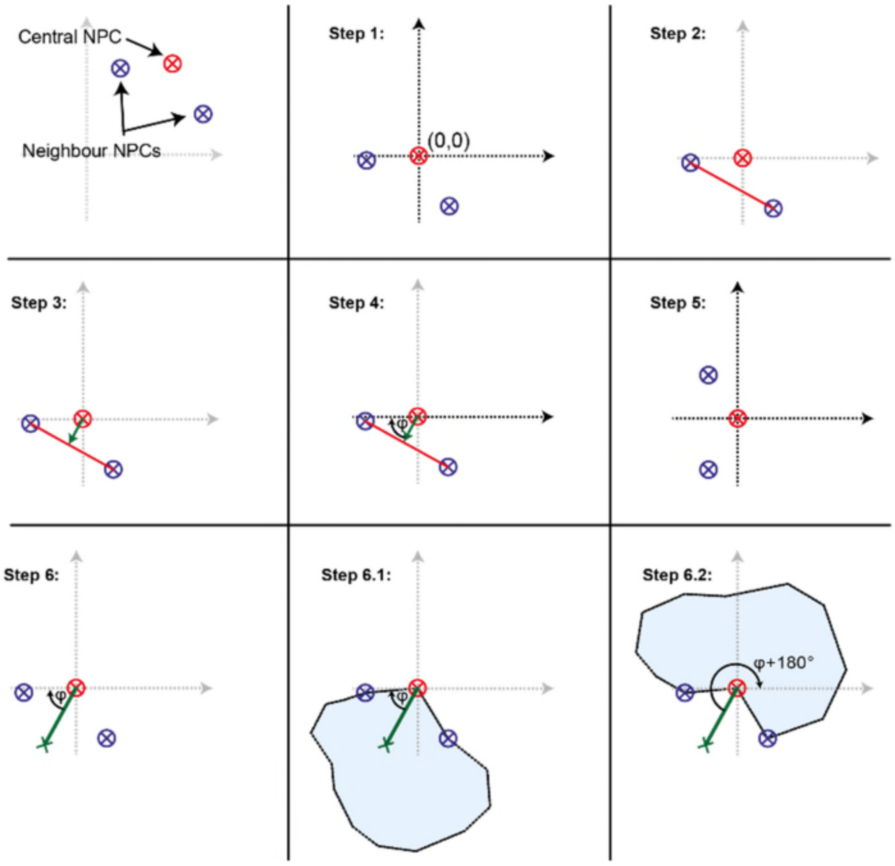

Supplemental Figure 11

Alignment of the export tracks onto the origin and the export direction to the abscissa. The coordinate system was shifted such that the transporting, "central" NPC (marked in red) represented the new origin. We assumed that export occurred along the axis, which was perpendicular to the connecting line between the two NPCs (blue marks), which were neighboured to the transporting, "central" NPC (marked in red). This line was parallel to the ordinate in Step 5. Usually, the NPCs formed a convex line (Step 6.1), but in rare cases the NE line was concave, which was considered by rotating the track coordinates by φ+180° (step 6.2).

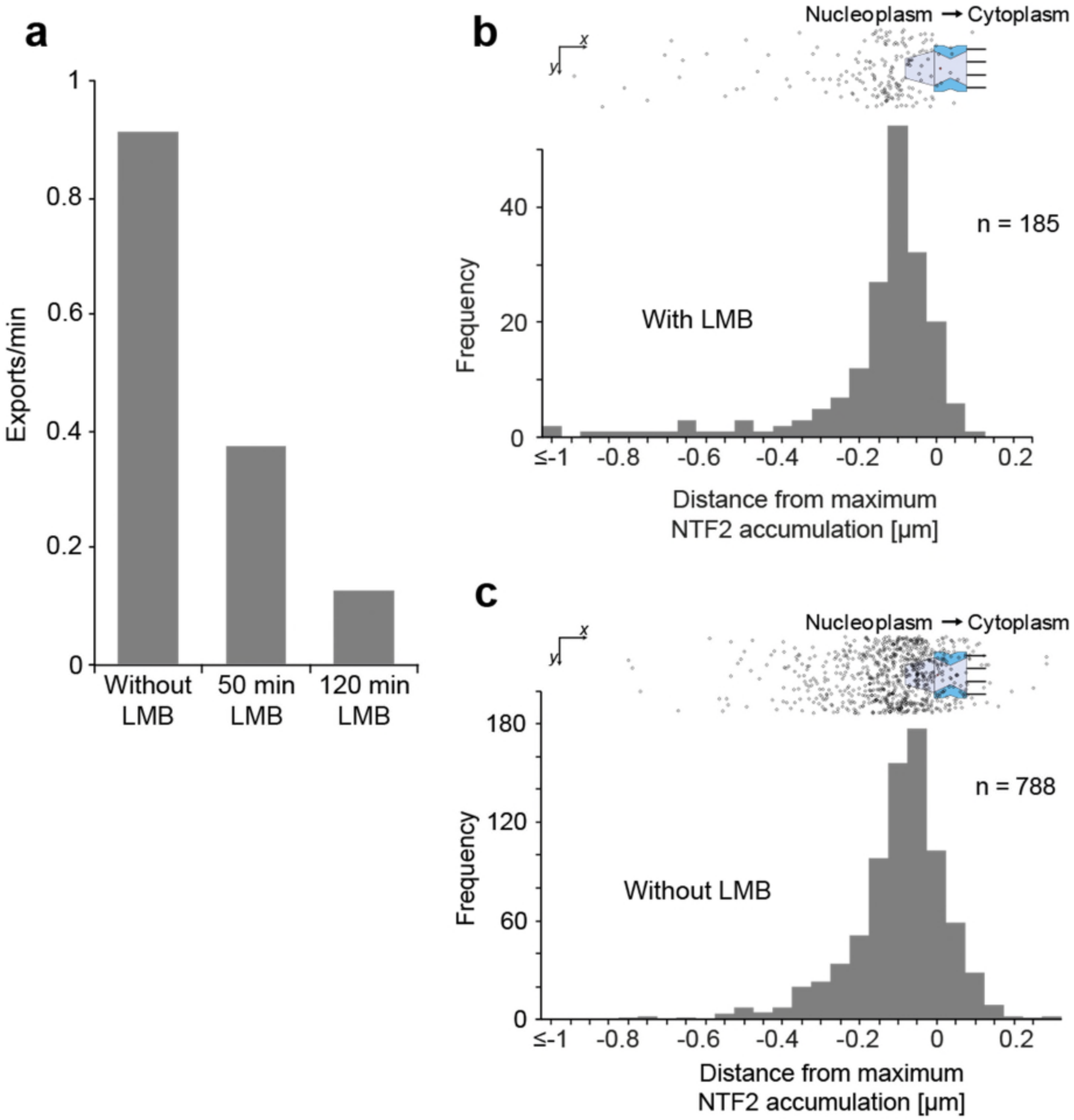

Supplemental Figure 12

Interaction of single pre-60S particles with NPCs in the presence of LMB. (a) Number of detected pre-60S export events in the absence, and 50 and120 after addition of LMB, respectively. Failed nuclear export of pre-60S particles (b) in the presence and (c) absence of LMB (identical to Fig. 5b). The number of total positions plotted in (b) was *n*=185. (Top) The trajectories were aligned to the transporting NPC (as indicated by the maximum of the eGFP-NTF2 signal) as origin and then rotated such that the export direction coincided with the positive *x*-axis. (Bottom) The number of pre-60S particle positions in a *y*-axis region ± 100 nm off the central NPC was plotted as a function of the location with regard to the average NTF2 accumulation. The maxima in (b) and (c) clearly coincided with the nuclear basket. For these experiments, we analyzed movies from 81 different cells with a total run time of 26,7 min (50 min after addition of LMB) and from 42 different cells with a total run time of 15,5 min (120 min after addition of LMB).

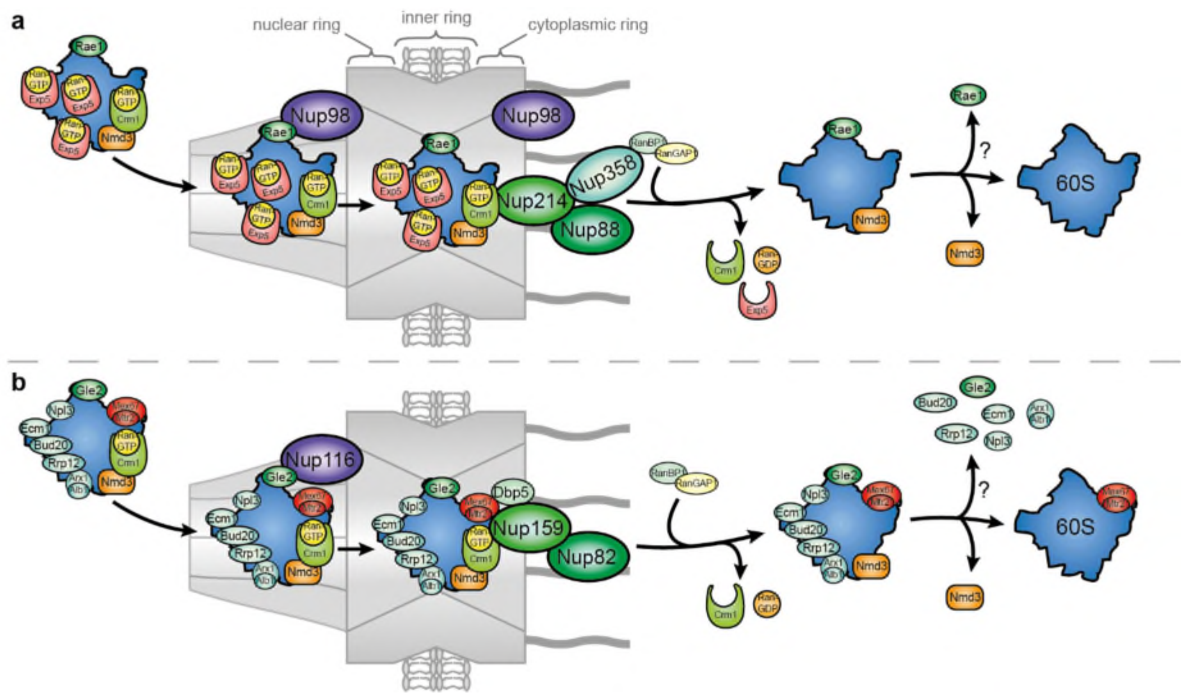

Supplemental Figure 13

Proposed pre-60S particle export mechanism in (a) human cells. Human pre-60S particle export begins with the non-FG interaction of RAE1 with Nup98. The entry into the FG-repeat domain of the NPC channel is mediated by Exp5 and Crm1. Release from this requires the pre-60S particle/CRM1/Nup214 interaction at the cytoplasmic face of the NPC, which “fishes” the particle out of the central NPC domain. Subsequently the pre-60S particle escapes into the cytosol after CRM1 release induced by RanBP1/RanGAP1, which is recruited to the NPC by Nup358. (b) In yeast the receptor-loaded pre-60S particle first transiently interacts with Nup116 at the nuclear basket via Gle2 before being transported into the inner ring in a Mex67/Mtr2-dependent manner. Release from this energetically favourable NPC compartment would then similarly require 'fishing out' the Crm1/pre-60S particle cargo through strong interactions with the asymmetric NUP159, which is part of the Nup82 subcomplex, and subsequent removal of Crm1 by RanBP1/RanGAP1 for cytoplasmic release of the pre-ribosomes.

Supplementary Movie 1

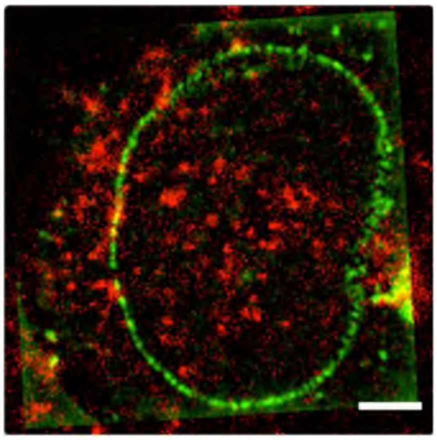

Supplementary Movie 2

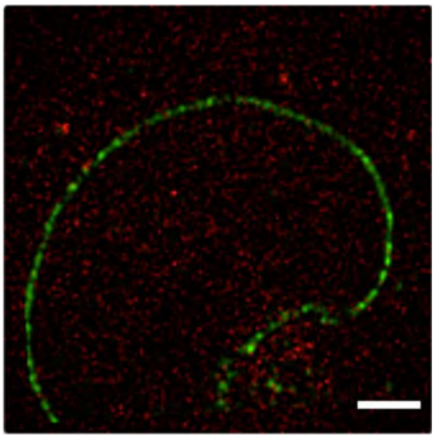

References cited in the supplemental material:

1. Gallo, S., Beugnet, A. & Biffo, S. Tagging of functional ribosomes in living cells by HaloTag® technology. *Vitr. Cell. Dev. Biol. - Anim.* **47**, 132–138 (2011).
2. Landvogt, L. *et al.* Observing and tracking single small ribosomal subunits in vivo. *Methods* **153**, 63–70 (2019).
3. Zemp, I. *et al.* Distinct cytoplasmic maturation steps of 40S ribosomal subunit precursors require hRio2. *J. Cell Biol.* **185**, 1167–1180 (2009).
4. Pool, M. R., Stumm, J., Fulga, T. A., Sinning, I. & Dobberstein, B. Distinct modes of signal recognition particle interaction with the ribosome. *Science* **297**, 1345–8 (2002).
5. Wyler, E. *et al.* The beta-isoform of the BRCA2 and CDKN1A(p21)-interacting protein (BCCIP) stabilizes nuclear RPL23/uL14. *FEBS Lett.* **588**, 3685–91 (2014).
6. Grote, M., Kubitscheck, U., Reichelt, R. & Peters, R. Mapping of nucleoporins to the center of the nuclear pore complex by post-embedding immunogold electron microscopy. *J. Cell Sci.* **108**, 2963–2972 (1995).
7. Chatel, G. & Fahrenkrog, B. Dynamics and diverse functions of nuclear pore complex proteins. *Nucleus* **3**, 162–171 (2012).
8. Ma, J., Goryaynov, A. & Yang, W. Super-resolution 3D tomography of interactions and competition in the nuclear pore complex. *Nat. Struct. Mol. Biol.* **23**, 239–47 (2016).
9. Bayliss, R. *et al.* Interaction between NTF2 and xFxFG-containing nucleoporins is required to mediate nuclear import of RanGDP. *J. Mol. Biol.* **293**, 579–93 (1999).
10. Iborra, F. J., Jackson, D. A. & Cook, P. R. The path of RNA through nuclear pores: apparent entry from the sides into specialized pores. *J. Cell Sci.* **113 Pt 2**, 291–302 (2000).
