# Supplementary material for: Nuclear export of the pre-60S ribosomal subunit through single nuclear pores observed in real time
Source: Nat Commun. 2021 Oct 27;12:6211. doi: 10.1038/s41467-021-26323-7 (PMC8551241; doi:10.1038/s41467-021-26323-7)
Supplement: Supplementary file 3 — Description of Additional Supplementary Files [file 41467_2021_26323_MOESM3_ESM.docx]

Description of Additional Supplementary Files

**Title: Supplementary Movie 1.**

**Description:** HeLa cell stably expressing GFP-NTF2 and eIF6-HaloTag labelled with JF549-HaloTag-Ligand. Single pre-60S particles (red) diffuse within a nucleus. The nuclear envelope is shown in green. Scale bar, 3 µm.

**Title: Supplementary Movie 2.**

**Description:** Single pre-60S particle (red) passing the nuclear envelope (green). The image data was smoothed and contrast-enhanced to improve the visualization. Scale bar, 3 µm.
