# Supplementary material for: Nuclear export of the pre-60S ribosomal subunit through single nuclear pores observed in real time
Source: Nat Commun. 2021 Oct 27;12:6211. doi: 10.1038/s41467-021-26323-7 (PMC8551241; doi:10.1038/s41467-021-26323-7)
Supplement: Supplementary file 6 — Reporting Summary [file 41467_2021_26323_MOESM6_ESM.pdf]

nature research

Corresponding author(s): Ulrich Kubitscheck

Last updated by author(s): Aug 17, 2021

Reporting Summary

Nature Research wishes to improve the reproducibility of the work that we publish. This form provides structure for consistency and transparency in reporting. For further information on Nature Research policies, see our Editorial Policies and the Editorial Policy Checklist.

Statistics

For all statistical analyses, confirm that the following items are present in the figure legend, table legend, main text, or Methods section.

n/a	Confirmed	
☐	☒	The exact sample size (n) for each experimental group/condition, given as a discrete number and unit of measurement
☐	☒	A statement on whether measurements were taken from distinct samples or whether the same sample was measured repeatedly
☒	☐	The statistical test(s) used AND whether they are one- or two-sided<br>*Only common tests should be described solely by name; describe more complex techniques in the Methods section.*
☒	☐	A description of all covariates tested
☒	☐	A description of any assumptions or corrections, such as tests of normality and adjustment for multiple comparisons
☐	☒	A full description of the statistical parameters including central tendency (e.g. means) or other basic estimates (e.g. regression coefficient) AND variation (e.g. standard deviation) or associated estimates of uncertainty (e.g. confidence intervals)
☒	☐	For null hypothesis testing, the test statistic (e.g. F, t, r) with confidence intervals, effect sizes, degrees of freedom and P value noted<br>*Give P values as exact values whenever suitable.*
☒	☐	For Bayesian analysis, information on the choice of priors and Markov chain Monte Carlo settings
☒	☐	For hierarchical and complex designs, identification of the appropriate level for tests and full reporting of outcomes
☒	☐	Estimates of effect sizes (e.g. Cohen's d, Pearson's r), indicating how they were calculated

Our web collection on statistics for biologists contains articles on many of the points above.

Software and code

Policy information about availability of computer code

Data collection	Airyscan confocal images were acquired with Zen Black (Zeiss, version 2.3 SP1). Single molecule measurements were acquired using SOLIS (Andor, version X-4603).
Data analysis	The following programs were used for data analysis: Fiji (Schindelin et al (2012), version 1.52p), Excel (Microsoft, version 2007 SP3), OriginPro 8 (OriginLab Corporation, version v8.0988), Mathematica (Wolfram, version 12.1.0.0), Eclipse IDE (Eclipse Foundation, Inc, version Photon release 4.8.0). All custom code is described in the Code and Software Submission Checklist. It can be downloaded from: https://www.chemie.uni-bonn.de/pctc/kubitscheck/downloads

For manuscripts utilizing custom algorithms or software that are central to the research but not yet described in published literature, software must be made available to editors and reviewers. We strongly encourage code deposition in a community repository (e.g. GitHub). See the Nature Research guidelines for submitting code & software for further information.

Data

Policy information about availability of data

All manuscripts must include a data availability statement. This statement should provide the following information, where applicable:

- Accession codes, unique identifiers, or web links for publicly available datasets
- A list of figures that have associated raw data
- A description of any restrictions on data availability

The datasets generated during the current study are available from the corresponding author on reasonable request. Source data are provided with this paper. As explained in the editorial requests file, the data sets generated during the current study comprise about 500 Gigabytes of multi-channel images. Images were taken of the same specimen regions using two different imaging devices (EMCCD and laser scanning microscope). The images had to be scaled and aligned with the help of reference beads. All this was in detail explained in the Methods and was achieved using complex custom-developed software routines. The pure data, even

annotated, are worthless without the appropriate evaluation procedure. Therefore it does not make sense to publish the data as such. We are happy to answer any reasonable request for the data and to provide access to the raw data and to explain and assist in the application of the data analysis procedures.

Field-specific reporting

Please select the one below that is the best fit for your research. If you are not sure, read the appropriate sections before making your selection.

- [x] Life sciences
- [ ] Behavioural & social sciences
- [ ] Ecological, evolutionary & environmental sciences

For a reference copy of the document with all sections, see nature.com/documents/nr-reporting-summary-flat.pdf

Life sciences study design

All studies must disclose on these points even when the disclosure is negative.

Sample size	We did not use predetermined sample sizes. Measurements were repeated n times and the results were averaged. This process was repeated until the standard error of the mean was less or equal 10% of the mean value.
Data exclusions	No data were excluded from the measurements.
Replication	Experiments were performed at least three times. All attempts to replicate were successful.
Randomization	Randomization was intrinsic, because we performed measurements on single cells, which were selected randomly from sample preparations each comprising hundreds of cells. Each single cell experiment was repeated at least three times on independent sample preparations.
Blinding	Blinding was not relevant to our study, because all experiments using single cells were evaluated using a complex data analysis procedure, which was extensively described in the Methods section. This procedure used in the same manner for all experiments and was therefore blind to a given experimental condition. The experimentalist had no means to influence the performance of the automatic procedure, which was identical for all different experiments. In other words, the results were determined by automatic routines.

Reporting for specific materials, systems and methods

We require information from authors about some types of materials, experimental systems and methods used in many studies. Here, indicate whether each material, system or method listed is relevant to your study. If you are not sure if a list item applies to your research, read the appropriate section before selecting a response.

Materials & experimental systems

n/a	Involved in the study	
[ ]	[x]	Antibodies
[ ]	[x]	Eukaryotic cell lines
[x]	[ ]	Palaeontology and archaeology
[x]	[ ]	Animals and other organisms
[x]	[ ]	Human research participants
[x]	[ ]	Clinical data
[x]	[ ]	Dual use research of concern

Methods

n/a	Involved in the study	
[x]	[ ]	ChIP-seq
[x]	[ ]	Flow cytometry
[x]	[ ]	MRI-based neuroimaging

Antibodies

Antibodies used

Immune blot:
- RPL10 Monoclonal Antibody (OTI6B11), #TA807662 (Lot: #VD2974632), Thermo Fisher
-I gG-HRP, #sc-2304 (Lot: #C221), Santa Cruz Biotechnology)

- NTF2 Monoclonal Antibody, #sc-271693 (Lot: #B1914), Santa Cruz Biotechnology
- eGFP Monoclonal Antibody F56-6A1.2.3, #MA1-952 (Lot: #TE260472), Thermo Fisher
- Goat anti-Mouse IgG (H+L) Secondary Antibody, HRP, #31430 (Lot: #UJ293428), Thermo Fisher

NTF2 localization:
- mAb414, MMS-120P (#902907), Covance (Biolegend)
- Anti-Mouse IgG (whole molecule)–Atto 488 antibody produced (goat), 62197 (Lot# BCCB9457), Sigma-Aldrich

Sucrose Gradient:
- NMD3 and RPS2 from Zemp, I. et al. Distinct cytoplasmic maturation steps of 40S ribosomal subunit precursors require hRio2. J. Cell Biol. 185, 1167–1180 (2009).
- RPL23A from Pool, M. R., Stumm, J., Fulga, T. A., Sinning, I. & Dobberstein, B. Distinct modes of signal recognition particle interaction with the ribosome. Science 297, 1345–8 (2002).

	- eIF6-HaloTag from Wyler, E. et al. The beta-isoform of the BRCA2 and CDKN1A(p21)-interacting protein (BCCIP) stabilizes nuclear RPL23/uL14. FEBS Lett. 588, 3685–91 (2014)
Validation	All antibodies were validated by Western blotting and immunofluorescence microscopy in our lab.

Eukaryotic cell lines

Policy information about cell lines

Cell line source(s)	The parental HeLa cell line was purchased from ATCC (Germany).
Authentication	None of the cell lines used were authenticated.
Mycoplasma contamination	All cell lines used were tested negative for mycoplasm.
Commonly misidentified lines (See ICLAC register)	No commonly misidentified cell line was used in this study.
