# Supplementary figures and images for: Nuclear export of the pre-60S ribosomal subunit through single nuclear pores observed in real time
Source: Nat Commun. 2021 Oct 27;12:6211. doi: 10.1038/s41467-021-26323-7 (PMC8551241; doi:10.1038/s41467-021-26323-7)

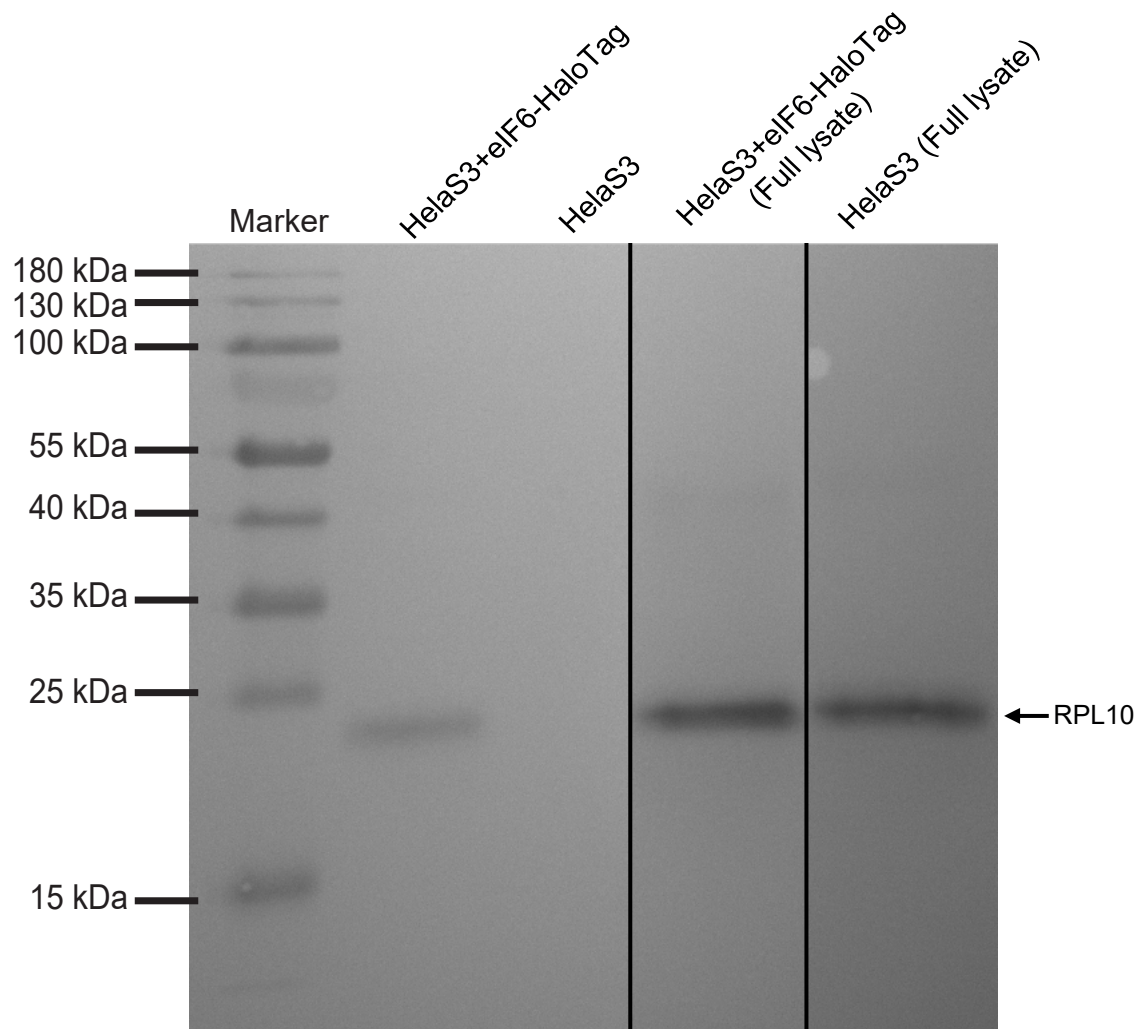

Supplement: Supplementary file 7 — Source Data [file 41467_2021_26323_MOESM7_ESM.zip › Figure-1-d.pdf]

uncropped blots supplemental figure 3d

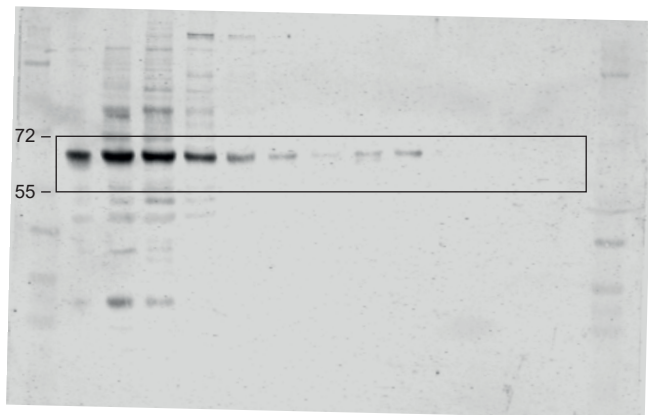

eIF6-Halo

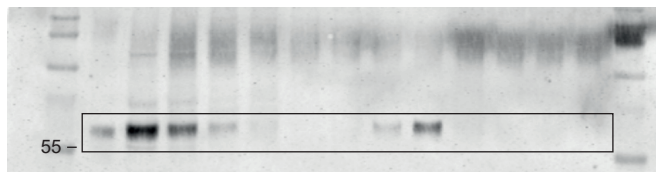

NMD3

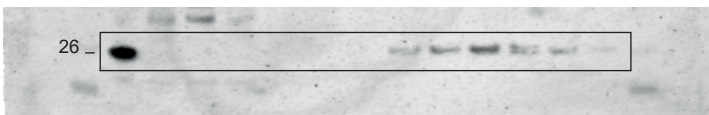

RPL23A

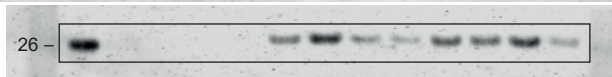

RPS3

Supplement: Supplementary file 7 — Source Data [file 41467_2021_26323_MOESM7_ESM.zip › Sup-Figure-3-d.pdf]

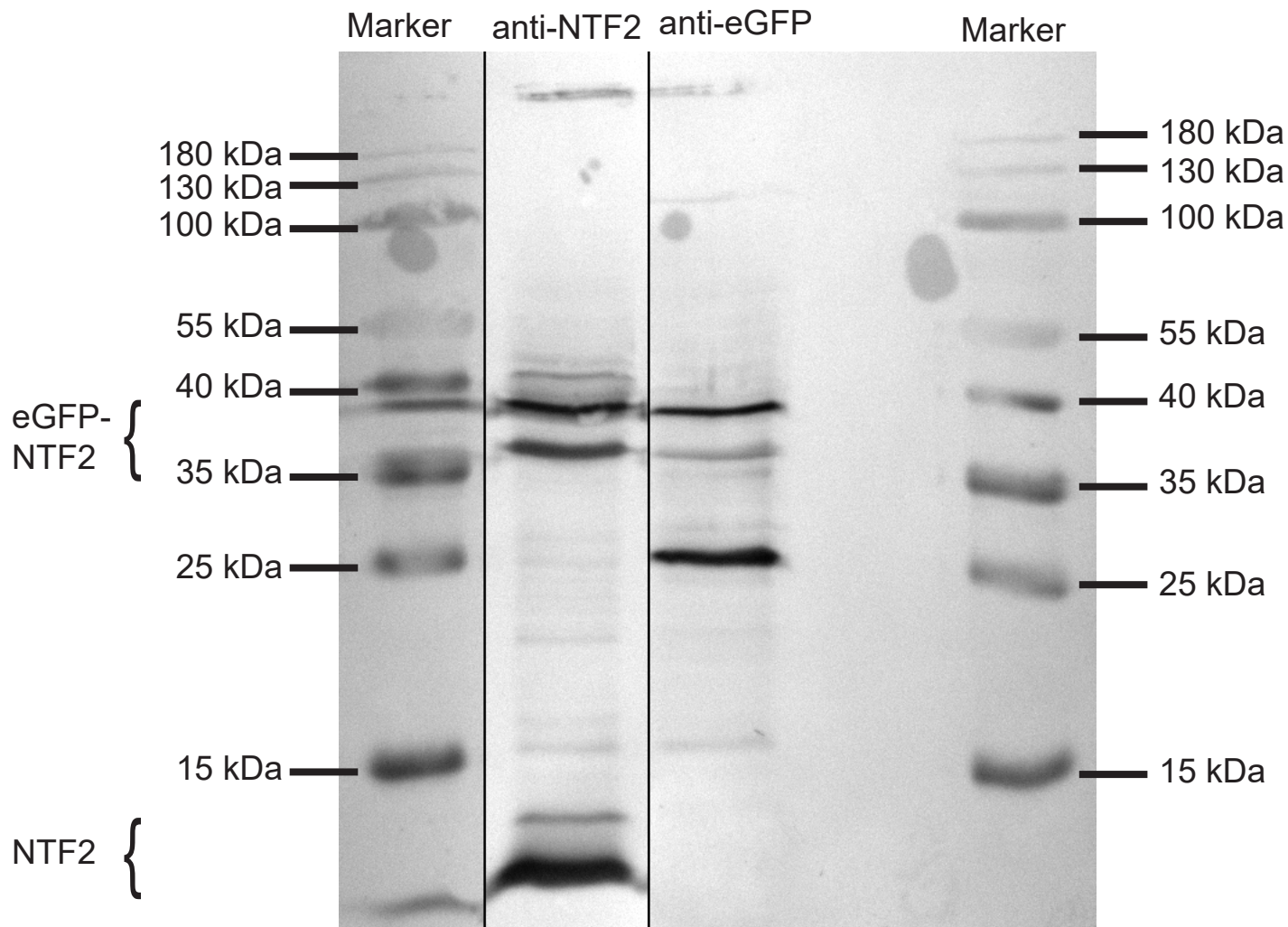

Supplement: Supplementary file 7 — Source Data [file 41467_2021_26323_MOESM7_ESM.zip › Sup-Figure-4-a.pdf]
